# Electrochemical Postfunctionalization of Polymers: Versatile Strategies for Macromolecular Transformation

**DOI:** 10.1021/acs.accounts.6c00464

**Published:** 2026-09-01

**Authors:** Kohei Taniguchi, Kosuke Sato, Shinsuke Inagi

**Affiliations:** Department of Chemical Science and Engineering, School of Materials and Chemical Technology, Institute of Science Tokyo, 4259 Nagatsuta-cho, Midori-ku, Yokohama 226-8501, Japan

## Abstract

Postsynthetic modification of polymers is a crucial strategy for tailoring macromolecular properties and achieving complex architectures that are typically inaccessible through the polymerization of available monomers alone. However, conventional chemical editing typically relies on stoichiometric amounts of hazardous oxidants or reductants, extreme temperatures, and heavy metal catalysts, presenting significant environmental and economic challenges. In recent years, the electrochemical transformation approach has emerged as a powerful, mild, and highly sustainable alternative. Because electrons serve as the sole redox reagent, electrochemical postfunctionalization operates under ambient conditions without generating stoichiometric waste, inherently aligning with the principles of green chemistry. This approach not only eliminates toxic reagents but also provides simple reaction protocols and enables precise control over the degree of functionalization by tuning the applied potential or current, thereby enabling systematic tailoring of macromolecular properties.

This review categorizes electrochemical postfunctionalization into four distinct mechanistic modes. The first mode involves direct activation of polymer films deposited on electrodes, where heterogeneous electron transfer initiates modifications. The second mode involves direct activation of dissolved polymers, enabling homogeneous transformations along the macromolecular chain. The third mode employs redox-mediated indirect activation, overcoming kinetic barriers and facilitating polymer backbone modification. Finally, the fourth mode involves electrochemically generated reactive species that migrate from the electrode to react with polymers in solution.

Based on these fundamental modes, we highlight the electrochemical postfunctionalization of conjugated polymers. A critical factor governing these transformations is the solubility of the oxidized (doped) polymer species, which determines whether the reaction proceeds in the solid state or in solution. Careful selection of appropriate reaction conditions enables highly efficient modifications, allowing precise tuning of the optical, structural, and electronic properties of the resulting polymers. These advances enable the development of next-generation organic electronic materials.

Furthermore, this review explores the application of electrochemical postfunctionalization to nonconjugated commodity plastics, including polystyrene, poly­(vinyl chloride), and poly­(vinylidene chloride), to address the global waste crisis. For these chemically inert materials, solution-phase electrochemical activation provides efficient and sustainable upcycling pathways. These strategies enable simultaneous deconstruction of persistent plastic waste and generation of value-added fine chemicals, thereby transitioning waste management toward chemical valorization.

This review demonstrates that electrochemical postfunctionalization provides a unified and sustainable platform for polymer modification, advancing both the development of functional materials and the realization of a circular plastics economy.

## Graphical abstract

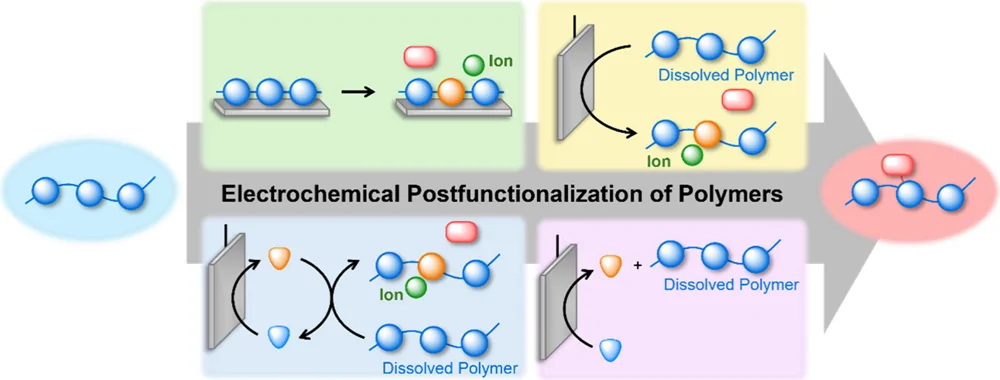

## Key References

-

Taniguchi, K. ; Kurioka, T. ; Sato, K. ; Tomita, I. ; Inagi, S. Electrochemical Postfunctionalization of Thiophene–Fluorene Alternating Copolymers via Anodic C–H Phosphonylation. Macromolecules 2024, 57­(10), 5028–5037 10.1021/acs.macromol.4c00465 .^1^ *This study reports the electrochemical postfunctionalization of conjugated polymers triggered by solid-to-solid electron transfer*.
-

Taniguchi, K. ; Sato, K. ; Inagi, S. Precisely Controlled Electrochemical Phosphonylation: Tailoring π-Conjugated Polymer Properties for High-Performance Organic Electrochemical Transistors. Angew. Chem., Int. Ed. 2026, 65 e1180643 10.1002/anie.1180643 41999336.^2^ *This study reports the precisely controlled electrochemical postfunctionalization of conjugated polymers and their application to organic electrochemical transistors*.
-

Taniguchi, K. ; Sato, K. ; Inagi, S. Electrochemical C–H Phosphonylation of Dissolved Conjugated Polymers via Direct Electron Transfer at the Electrode. Macromolecules 2025, 58­(19), 10526–10535 10.1021/acs.macromol.5c01783 .^3^ *This study reports the electrochemical functionalization of dissolved conjugated polymers, which are activated via solution-phase electron transfer at the electrode*.
-

Wang, S. ; Taniguchi, K. ; Motokawa, K. ; Sato, K. ; Inagi, S. Electrochemical Postmodification of Polystyrene via Aromatic C–H Iodination. ACS Macro Lett. 2025, 14­(10), 1615–1621 10.1021/acsmacrolett.5c00585 41077765.^4^ *This study reports the postfunctionalization of polystyrene using electrochemically generated reagents*.

## 1 Introduction

Electrochemistry has emerged as a transformative tool in organic synthesis, providing novel pathways for selective chemical transformations.^5–11^ Over the past decade, electrosynthesis has evolved from a niche technique into a general strategy for chemical modification.^12–14^ Applying an appropriate potential to an electrode induces electron transfer with substrates, thereby generating activated species. Subsequently, these intermediates undergo various chemical transformations, such as nucleophilic, electrophilic, and radical reactions.

In electrochemical reactions, electrons serve as mass-free oxidants and reductants; consequently, cost-effective, sustainable, and mild reaction conditions can be achieved. Unlike conventional chemical redox reactions, which typically require stoichiometric reagents, high temperatures, or elevated pressures, electrochemical methods operate under ambient conditions without external chemical oxidants or reductants, making them simpler and less hazardous. Furthermore, if the electricity is derived from renewable energy sources, such as solar or wind power, the sustainability profile of the electrochemical reaction is enhanced.

Moreover, electrochemical reactions offer precise control through the simple tuning of electrochemical parameters in contrast to the careful selection of appropriate oxidants or reductants and their stoichiometry in conventional chemical approaches.^15^ For instance, constant-potential electrolysis improves the selectivity of desired redox reactions, whereas constant-current electrolysis allows direct control over the reaction rate.^16^ Therefore, selecting the appropriate electrolysis method and conditions enables highly selective synthesis of target products.

By leveraging these advantages, polymer chemists have developed electrochemical methods for polymer modification driven by electron transfer at electrodes.^17–20^ These approaches encompass four types of electrochemical postfunctionalization (*e*PF) (Figure 1). Polymer films deposited on electrodes can be activated via direct electron transfer to undergo chemical reactions;^19^ this mode is ideal for conjugated polymer (CP) films, which possess inherent electronic conductivity (Figure 1a). Alternatively, dissolved polymers can be directly activated by electron transfer at the electrode surface to participate in electrochemical reactions (Figure 1b).^17^ Furthermore, dissolved polymers can be activated indirectly via redox mediators that are oxidized or reduced at the electrode surface (Figure 1c), enabling polymer modification without direct contact between the polymer and the electrode surface. In addition, electrochemically generated reactive species can react with dissolved polymers, providing additional pathways for solution-phase functionalization (Figure 1d). In this article, these reaction modes are introduced and discussed.

**Figure 1:**
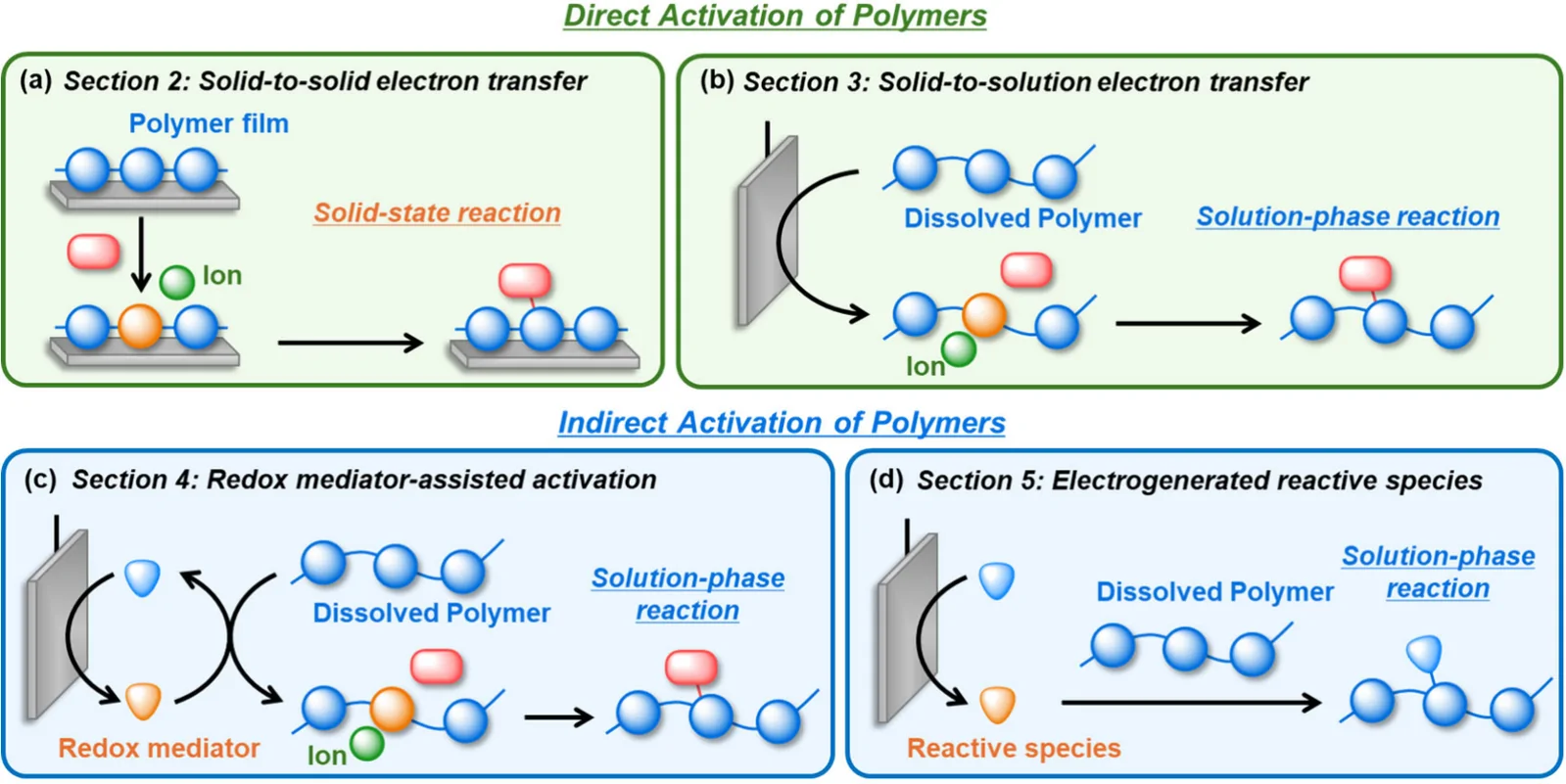
Schematic representation of *e*PF modes. Direct activation type: (a) solid-to-solid and (b) solid-to-solution electron transfer. Indirect activation type: (c) redox-mediator-assisted polymer activation and (d) electrochemically generated reactive species for polymer reaction.

## 2 Functionalization of Polymers Induced by Solid-to-Solid Electron Transfer

In this mode, CP films deposited on the electrode are directly activated via solid-to-solid electron transfer, enabling solid-state *e*PF. This approach requires inherent electronic conductivity in the polymer film and is therefore best suited to CPs. Electrochemical oxidation or reduction alters the redox state of CPs from their neutral form to a charged (doped) state. When a CP film is deposited on an electrode, and an appropriate potential is applied, direct electron transfer occurs between the polymer and the electrode, yielding radical ionic species (polarons) or dicationic species (bipolarons) within the extended π-conjugated backbone, modulating the optical properties and electrical conductivity of the CP film.^21–24^ To maintain electroneutrality, counterions (dopants) from the electrolyte are incorporated into the film. In contrast, applying an opposite potential converts the doped polymer back to its neutral state, accompanied by the release of the incorporated ions (dedoping). This reversible cycle is fundamental for controlling organic electronic devices,^24–27^ where maintaining a doped state ensures operational stability.

From the perspective of synthetic chemistry, a doped state can be regarded as a reactive intermediate that enables subsequent chemical transformations. For example, when poly­(3-hexylthiophene) (P3HT) is oxidized, cationic species are generated along the π-conjugated backbone, rendering the polymer susceptible to nucleophilic attacks. Overall, electrochemical polymer modification is cost-effective, sustainable, and experimentally simple.^19,28^ Furthermore, the reaction can be accurately tuned by controlling the amount of charges passed (i.e., Faradaic charge), enabling fine control over the degree of functionalization (DOF, the fraction of functionalized repeating units within the polymer chain) of CP films.^19,28^

The pioneering study on *e*PF involved the anodic chlorination of poly­(3-methylthiophene) (P3MT) reported by Qi and Pickup.^29^ A P3MT film was first prepared via the electrochemical polymerization of 3-methylthiophene monomers. The resulting P3MT-coated electrode was then subjected to anodic oxidation in an acetonitrile (MeCN) solution containing 0.1 mol L^–1^ tetraethylammonium chloride (Et_4_NCl) used as the supporting electrolyte and chloride source. Cl^–^ ions were incorporated into the P3MT film via electrochemical doping; these ions then acted as nucleophiles, attacking the doped polymer backbone. This process was followed by further oxidation and deprotonation, resulting in the formation of C–Cl bonds at the 4-position of the thiophene repeating units. Electron microprobe analysis confirmed the quantitative chlorination of the thiophene rings, demonstrating successful C–Cl bond formation (Table 1 entry 1).

**Table 1:**
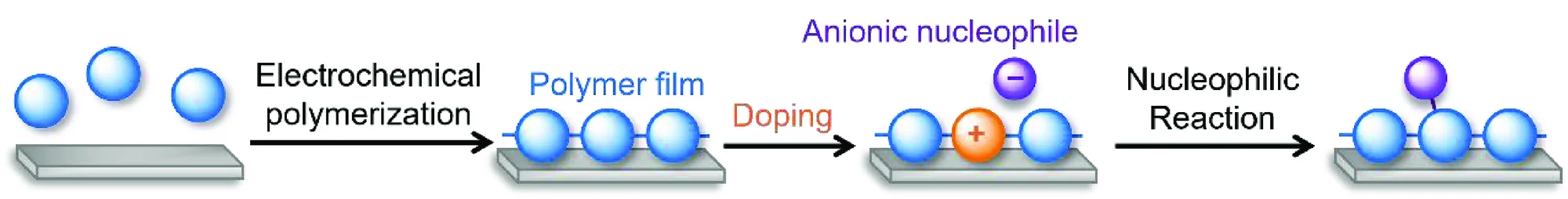

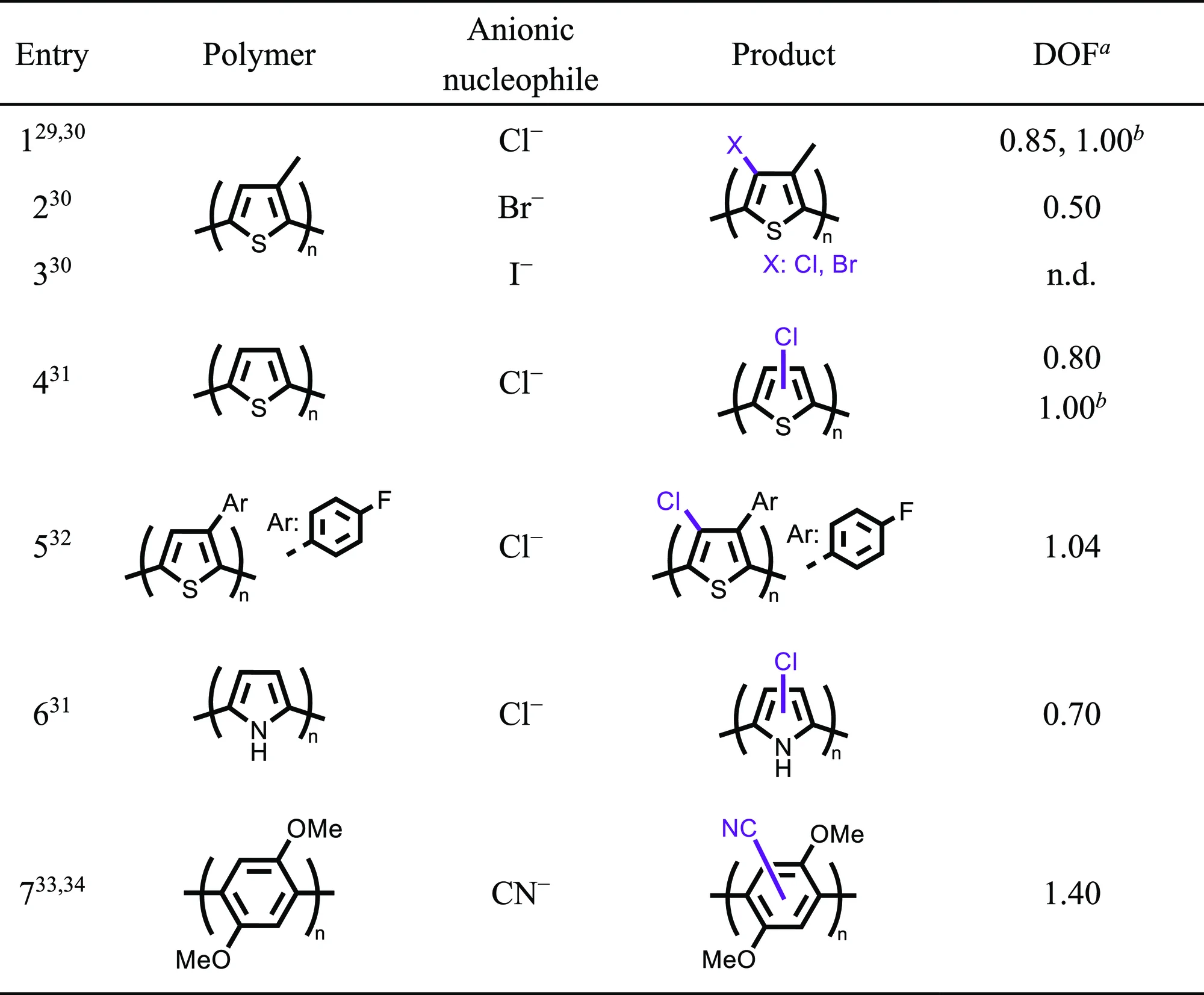
Anodic Functionalization of CP Film Obtained via Electrochemical Polymerization^a^ ^,^ ^b^

a DOF was determined using electron microprobe analysis or XPS.

b Repeated procedure was performed.

Subsequently, Qi and Pickup achieved the anodic bromination of P3MT using 0.1 mol L^–1^ tetrabutylammonium bromide instead of Et_4_NCl (Table 1 entry 2), whereas anodic iodination using 0.1 mol L^–1^ tetrabutylammonium iodide was unsuccessful (Table 1 entry 3).^30^ Electron microprobe analysis revealed degrees of chlorination and bromination of approximately 0.85 and 0.50, respectively, for the P3MT precursor films. The repeated application of this procedure increased the DOF to 1.00 (Table 1 entry 4). Pickup and colleagues also reported the anodic chlorination of polythiophene and polypyrrole, achieving DOFs of 0.80 and 0.70, respectively (Table 1 entries 4,6).^31^ Subsequently, Bélanger et al. demonstrated the anodic chlorination of poly­(3-(4-fluorophenyl)­thiophene) and reported that the phenyl substituent of the thiophene unit did not prevent chlorination on the 4-position of the thiophene (Table 1 entry 5). Furthermore, the reverse reaction, namely, the cathodic dechlorination of the chlorinated polymer, was successfully achieved.^32^ These reversible processes were confirmed by electrochemical analysis, energy-dispersive X-ray spectroscopy, and X-ray photoelectron spectroscopy (XPS). Notably, XPS analysis revealed that the oxidation state of sulfur in the thiophene rings remained unchanged during reversible chlorination and dichlorination.^32^

Fabre and colleagues demonstrated the anodic cyanation of poly­(*p*-dimethoxybenzene) in MeCN containing 0.2 mol L^–1^ tetrabutylammonium cyanide.^33,34^ A poly­(*p*-dimethoxybenzene) film was first deposited onto an electrode via the electrochemical polymerization of 1,4-dimethoxybenzene. Then, the resulting polymer-coated electrode was used as the working electrode for anodic functionalization. The incorporation of cyano groups into the polymer was confirmed by Fourier transform infrared (FTIR) spectroscopy and XPS analysis. Based on XPS quantification, a DOF of 1.4 was achieved (Table 1 entry 7).

Based on the concept of *e*PF, *p*-doped CPs can react with nucleophilic dopant anions and charge-neutral (electrically neutral) nucleophiles. In general, the use of non-nucleophilic counterions, such as hexafluorophosphate and tetrafluoroborate, enables stable electrochemical doping of CP films. This observation motivates the investigation of whether the chemical structures of CPs can be transformed via doping in the presence of charge-neutral nucleophiles (Figure 2a).

**Figure 2:**
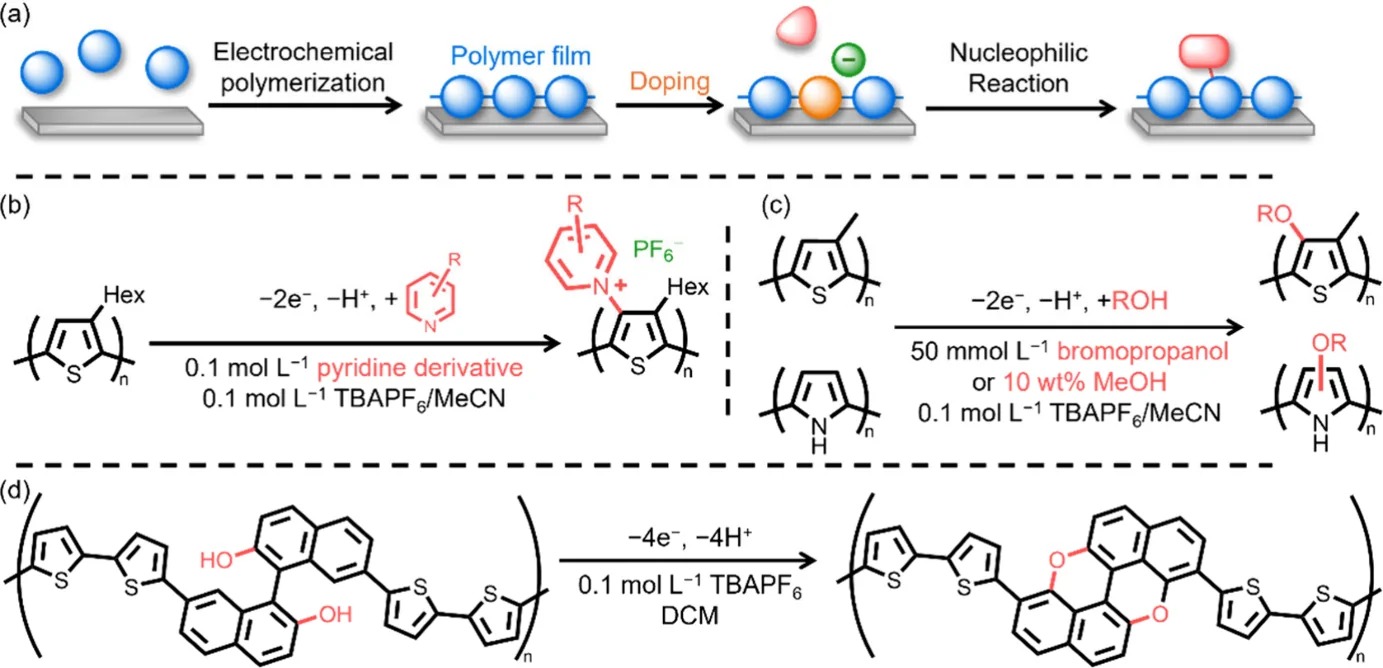
(a) Schematic representation of oxidative *e*PF with charge-neutral nucleophiles using electrochemically polymerized CP films. (b) Anodic pyridination of P3HT. (c) Anodic alkoxylation of P3MT and polypyrrole. (d) Anodic intramolecular cyclization of binaphthol-based CP film.

Iyoda et al. reported the anodic pyridination of electropolymerized P3HT films in MeCN containing tetrabutylammonium hexafluorophosphate (Bu_4_NPF_6_) as the supporting electrolyte and pyridine derivatives as charge-neutral nucleophiles (Figure 2b).^35^ The introduction of pyridinium moieties into the P3HT backbone was confirmed by FTIR spectroscopy and spectroelectrochemical measurements, and the DOF was 0.12 per repeating unit (corresponding to 0.60 per oxidized unit). Notably, the incorporation of these pyridinium moieties deactivated the p-type semiconductor properties.

Pickup and colleagues achieved the alkoxylation of electropolymerized P3MT and polypyrrole using tetraethylammonium perchlorate as the supporting electrolyte and methanol and bromopropanol as charge-neutral nucleophiles (Figure 2c).^31^ Bromopropanol was incorporated into the main chains with DOF values of 0.25 and 0.30 for P3MT and polypyrrole, respectively. MeOH could also be applied to the reaction, resulting in quantitative incorporation of methoxy groups.

Oxidative *e*PF can also proceed via internal nucleophilic functionalities. For instance, Swager and Song reported the oxidative intramolecular cyclization of an electropolymerized binaphthol-based CP film into a *peri*-xanthenoxanthene scaffold under high anodic potentials (Figure 2d).^36^ Validated by small-molecule models and *in situ* conductivity measurements, this transformation yielded more planar, extended π-conjugated motifs, resulting in a 2-fold increase in electrical conductivity.

Although *e*PF of electropolymerized CP films yields functionalized CPs, both the precursor and modified polymers are generally insoluble in common organic solvents, which limits characterization to solid-state techniques. Such analyses typically provide insufficient structural information, which is a drawback for the broader use of *e*PF of electropolymerized CP films as a universal postfunctionalization strategy. To address this limitation, *e*PF has been extended to chemically synthesized CPs that are soluble in common organic solvents, enabling detailed characterization using standard solution-based methods, such as nuclear magnetic resonance (NMR) and gel permeation chromatography (GPC).

Based on this concept, Inagi, Fuchigami, and colleagues synthesized soluble CPs (Figure 3) via Suzuki-Miyaura cross-coupling polymerization,^37,38^ chemical oxidative polymerization,^39^ and Grignard-metathesis polymerization.^40^ These CPs were soluble in common organic solvents, such as dichloromethane (CH_2_Cl_2_) and chloroform (CHCl_3_); however, they were insoluble in poor solvents, such as MeCN. The CP-dissolved CHCl_3_ solution was cast onto a platinum plate electrode, which was then used as the working electrode (Figure 3a). After the *e*PF of the CP film and its subsequent dedoping, the resulting polymer remained soluble in CHCl_3_ and CH_2_Cl_2_, indicating that it could still be analyzed by solution NMR and GPC. For instance, Inagi, Fuchigami, and colleagues demonstrated the electrochemical fluorination,^41,42^ carbonyl reduction,^43^ and parallel electrochemical oxidation and reduction of polyfluorene derivatives^44^ while preserving their solubility. These reactions have been discussed in previous reviews.^19,45^

**Figure 3:**
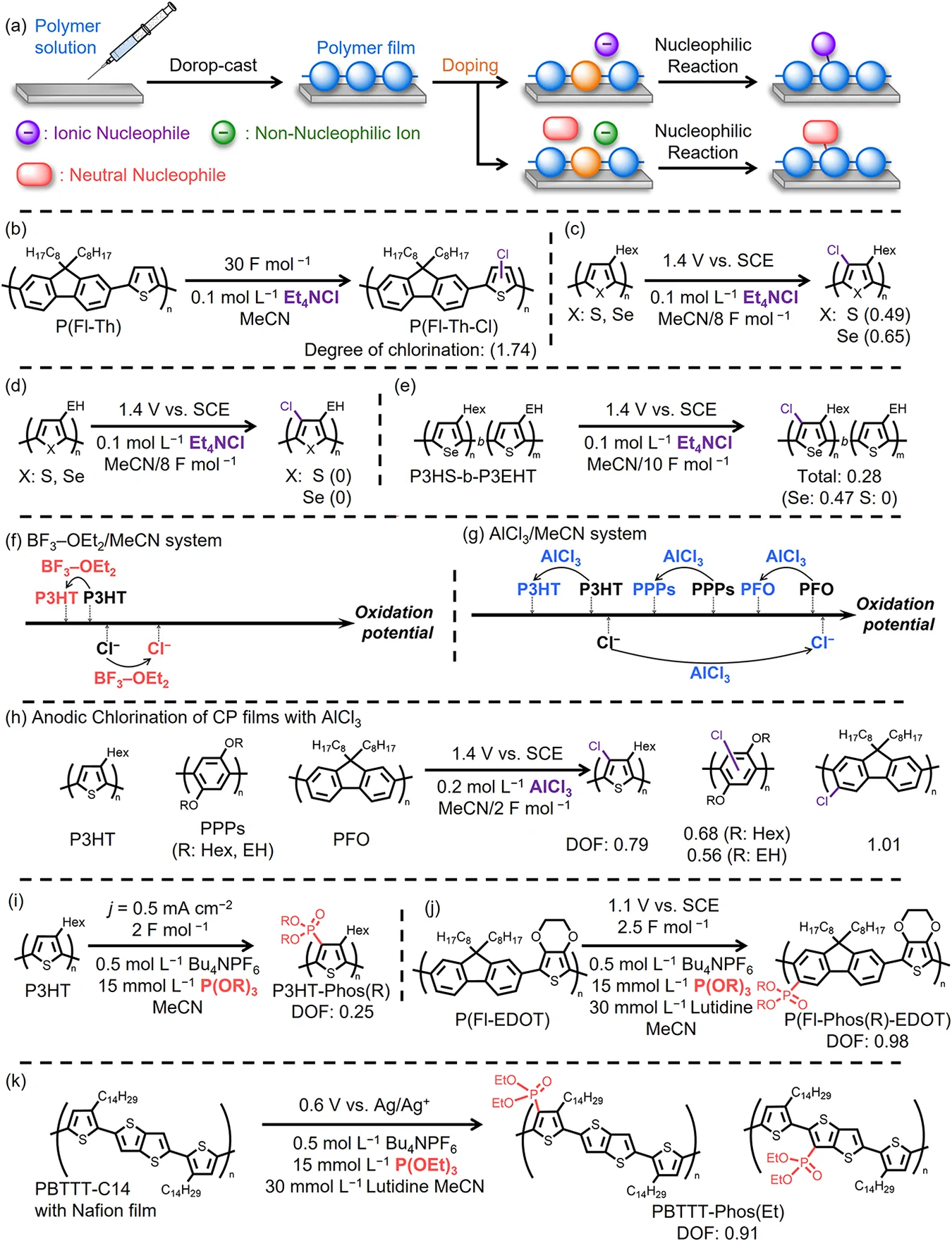
(a) Schematic representation of solid-state *e*PF of drop-cast CP films. Anodic chlorination of (b) P­(Fl-Th), (c) P3HT and P3HS, (d) P3EHT and P3EHS, and (e) P3HS-*b*-P3EHT. Adapted with permission from ref 37. Copyright 2009 American Chemical Society. Copyright 2009 Elsevier. Adapted with permission from ref 40. Copyright 2017 Wiley-VCH. Anodic chlorination of P3HT promoted by (f) BF_3_–OEt_2_ and (g) AlCl_3_. (h) Anodic chlorination of PPPs and PFO using the AlCl_3_/MeCN system. Reproduced with permission from ref 48. Copyright 2021 American Chemical Society. Anodic phosphonylation of (i) P3HT, (j) P­(Fl-EDOT), and (k) semicrystalline CP films using Nafion-composite films. Adapted with permission from refs 1, 2, and 52. Copyright 2022 Springer Nature; Copyright 2024 American Chemical Society; Copyright 2026 Wiley-VCH. The numbers in parentheses represent the DOF.

Fluorene–thiophene alternating copolymer (P­(Fl-Th)) was successfully subjected to anodic chlorination, yielding a DOF of 1.74, as determined by ^1^H NMR (Figure 3b).^37,38^ The ^1^H NMR spectrum of the chlorinated P­(Fl-Th) showed a decrease in the intensity of the signals derived from aromatic protons on thiophene rings compared with pristine P­(Fl-Th), indicating the substitution of these protons by chlorine atoms. Inagi and colleagues also reported the anodic chlorination of poly­(3-alkylchalcogenophene) derivatives, including P3HT, poly­(3-hexylselenophene) (P3HS), and block copolymers composed of their analogues.^39,40^ The DOFs were 0.49 and 0.65 for P3HT and P3HS, respectively (Figure 3c). Because P3HS typically has a lower highest occupied molecular orbital level than P3HT due to the heavier chalcogen atoms,^46^ it exhibits a higher chlorination level under the same conditions. XPS analysis of P3HS before and after chlorination confirmed C–Cl bond formation, indicating no change in the selenium oxidation state. Notably, replacing the hexyl side chain with a 2-ethylhexyl group, as observed in poly­(3-(2-ethylhexyl)­thiophene) (P3EHT) and poly­(3-(2-ethylhexyl)­selenophene) (P3EHS), substantially suppressed C–Cl bond formation during anodic chlorination (Figure 3d). These results indicate that reactivity toward anodic chlorination strongly depends on both the incorporated heteroatoms and the side-chain structure. The higher chlorination reactivity of P3HS than that of P3HT was maintained across different material formats, including block copolymers, statistical copolymers, and polymer blends (Figure 3e).

Although anodic chlorination is a useful and straightforward strategy, its current efficiency is typically limited by the undesired anodic oxidation of Cl^–^ ions.^19^ To improve current efficiency, Inagi, Kurioka, and colleagues reported that Lewis acids, such as boron trifluoride–diethyl ether (BF_3_–OEt_2_) and aluminum chloride (AlCl_3_), promote anodic chlorination of CPs (Figure 3f,g).^47,48^ The oxidation onset potential of P3HT in BF_3_–OEt_2_ shifted to more negative values than the onset potential observed in MeCN containing 0.1-mol L^–1^ tetraethylammonium tetrafluoroborate (Figure 3f). Moreover, BF_3_–OEt_2_ suppressed the anodic oxidation of Cl^–^ ions. Under these conditions, the anodic chlorination of P3HT in 0.1-mol L^–1^ Et_4_NCl/BF_3_–OEt_2_ yielded a DOF of 0.70 at 8 F mol^–1^ with enhanced current efficiency, consistent with the facilitated oxidation of P3HT and suppression of the competing Cl^–^ oxidation pathway.^47^

Furthermore, AlCl_3_ is particularly useful because it functions simultaneously as a Lewis acid, a supporting electrolyte, and a chlorine source in the AlCl_3_/MeCN system. This system facilitates the anodic oxidation of CPs and extends the electrochemical window (Figure 3g). Encouraged by these results, the anodic chlorination of CPs, such as P3HT, poly­(*p*-phenylene) derivatives, and poly­(9,9-dioctylfluorene) (PFO) in 0.2-mol L^–1^ AlCl_3_/MeCN under constant-current conditions was performed (Figure 3h).^48^ Compared with the BF_3_–OEt_2_ system, which yielded P3HT with a DOF of 0.70 at 8 F mol^–1^, the AlCl_3_/MeCN system provided more highly chlorinated P3HT (DOF: 0.79) at 2 F mol^–1^. These results highlight the superiority of the AlCl_3_/MeCN system for efficient anodic chlorination. In addition, alternating π-conjugated copolymers containing a 9,9-dioctylfluorene unit and another arylene unit (P­(Fl-Ar)) can participate in anodic chlorination using the 0.2-mol L^–1^ AlCl_3_/MeCN system.^49^ In these reports, GPC analysis revealed that CP degradation was negligible, indicating the preservation of the backbone.

Crystallinity plays a crucial role in anodic chlorination. The crystallinity of regioregular poly­(3-alkylthiophene) films is strongly influenced by the solvent used for drop-casting.^50^ For example, tetrahydrofuran promotes the formation of crystalline domains in P3EHT films, whereas CHCl_3_ and chlorobenzene (PhCl) yield amorphous films.^51^ Thus, the amorphous P3EHT film exhibited no reaction, whereas the crystalline P3EHT films yielded a DOF of 0.33.^51^

In addition, charge-neutral nucleophiles can participate in electrochemical polymer film reactions. Inagi, Taniguchi, and colleagues reported the anodic phosphonylation of P3HT with a 0.1 mol L^–1^ Bu_4_NPF_6_/MeCN solution containing 15 mmol L^–1^ trialkyl phosphite (P­(OR)_3_), yielding a DOF of up to 0.25 (Figure 3i).^52^ Furthermore, when fluorene–thiophene alternating copolymers were used as the substrate, the addition of 2,6-lutidine increased the DOF from 0.26 to 0.98 (Figure 3j).^1^ In addition, anodic phosphonylation could be applied to semicrystalline CP films using Nafion-composite films (Figure 3k), enabling near-quantitative phosphonylation (DOF: 0.91).^2^ In this system, Nafion decreases the size of crystalline domain of CP, promoting reagent diffusion within the film. Besides, the ionic sites of Nafion facilitate dopant transport. These effects lower the overpotential required for polymer oxidation and significantly improve the DOF compared with neat CP films. Notably, moderately phosphonylated samples (DOF = 0.08–0.16) exhibited improved organic electrochemical transistor performance when used as active-layer materials. These reports demonstrate that charge-neutral molecules can serve as effective functionalization reagents for *e*PF.

## 3 *e*PF of Dissolved Polymers Induced by Direct Electron Transfer at an Electrode

In this mode, dissolved polymers are directly activated via solid-to-solution electron transfer at the electrode surface, enabling solution-phase *e*PF. This approach is applicable to both conjugated and nonconjugated polymers, provided sufficient mass transport to the electrode is ensured. Whereas the solid-state *e*PF discussed in Section 2 has been applied exclusively to CPs, solid-to solution electron transfer extends the scope of *e*PF to nonconjugated polymers such as polystyrene and poly­(vinyl chloride) (PVC) (Figure 4a).

**Figure 4:**
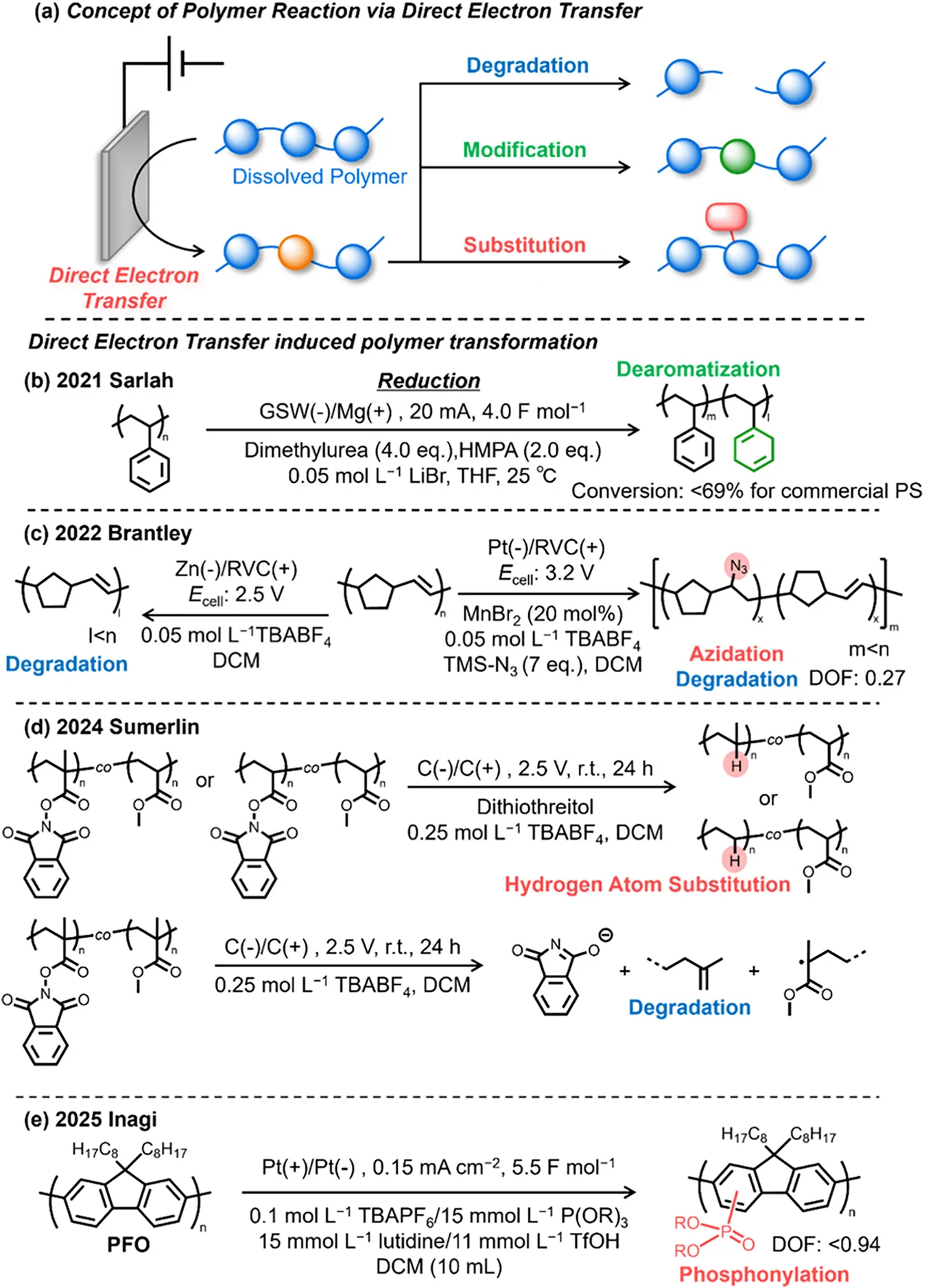
(a) Schematic representation of *e*PF via direct electron transfer at electrode. (b) Reductive dearomatization of polystyrene. (c) Electrochemical degradation and azidation of olefin-containing polymers. (d) Electrochemical hydrogenation and degradation of phthalimide-containing polymers via cathodic electron transfer. Adapted with permission from ref 56. Copyright 2024 Wiley-VCH. (e) Anodic phosphonylation of PFO. Adapted with permission from ref 3. Copyright 2025 American Chemical Society.

Based on this concept, several electrochemical transformations of nonconjugated polymers have been developed. Sarlah et al. reported that dearomatization of polystyrene derivatives occurs via electron transfer predominantly at the cathode–solution interface as polystyrene segments reach the electrode surface (Figure 4b).^53^ Consistent with small-molecule models,^54^ the electrochemical Birch reduction of simple arenes proceeds via direct electron transfer from the cathode to the arene, rather than via electron transfer from deposited Li^0^ to the substrate. Consistent with this view, polystyrene modification is proposed to follow a similar interfacial mechanism. This interpretation is also supported by control experiments, where chemical Birch and Benkeser conditions do not yield the corresponding dearomatized products.

Brantley et al. investigated the electrochemical degradation of ring-opening metathesis polymerization-derived polymers and demonstrated a mild electrochemical strategy for backbone editing of olefin-containing polymers (Figure 4c).^55^ Upon anodic oxidation, the polymer underwent β-scission, resulting in a decrease in molecular weight. In the presence of trimethylsilyl azide, the resulting radical chain ends were trapped to install azido functionalities. Notably, this approach selectively edits olefin-containing backbones, even within polymer mixtures, leaving nonolefinic components largely intact.

Sumerlin et al. reported the direct electron-transfer-initiated cathodic transformation of polymers containing *N*-(acryloxy)­phthalimide units (Figure 4d).^56^ At the cathode, the phthalimide redox-active ester undergoes irreversible one-electron reduction, followed by N–O bond cleavage to generate an acyloxy (carboxyl) radical that rapidly decarboxylates, yielding carbon-centered radicals on the polymer backbone. In the presence of a hydrogen-atom donor, such as dithiothreitol, these radicals were quenched to form C–H bonds (hydrogen-atom substitution). Furthermore, the ^1^H NMR spectrum of the product showed the absence of phthalimide protons at 7.7–8.0 ppm and a new signal at 1.2 ppm, indicating quantitative hydrogen-atom substitution. When a methacrylate copolymer containing PhthMA was subjected to electrolysis, polymer degradation was observed. The degradation was attributed to the β-scission of the backbone radicals generated upon decarboxylation, resulting in main-chain cleavage. Under optimized conditions, the degradation efficiency reached 96%, as determined by size-exclusion chromatography. In addition, Sumerlin and colleagues recently reported that block copolymers bearing tetrachlorophthalimide esters (ClPth­(M)­A) and tetrahydrophthalimide esters (HPth­(M)­A) exhibit distinct reduction potentials (e.g., ClPth­(M)­A at – 2.3 V vs HPth­(M)­A at – 2.5––3.0 V).^57^ Depending on the neighboring repeat unit, methacrylate-adjacent radicals preferentially undergo β-scission (degradation), whereas acrylate-adjacent radicals are predominantly capped via hydrogen-atom substitution.

Inagi and colleagues reported that CPs can undergo anodic phosphonylation in solution via direct electron transfer at the anode (Figure 4e).^3^ Despite the diffusion coefficient of PFO being approximately 25-fold smaller than that of small-molecule compounds, quantitative functionalization was achieved at low current densities (DOF up to 0.94). High current densities oxidize P­(OR)_3_, resulting in competitive electron transfer between PFO and P­(OR)_3_. This competition resulted in lower DOF values. Furthermore, the researchers demonstrated that sufficient mass transport, achieved through stirring-induced convection, is critical for solution-phase *e*PF of CPs, eliminating the need to predeposit a polymer film on the electrode.

Polymers incorporating side-chain redox-active moieties have been widely used in redox flow batteries and as macromolecular mediators.^58–60^ Francke and colleagues used TEMPO-modified polymethacrylates (TPMAs) as soluble, recyclable redox mediators (polymediators) for indirect electrochemical synthesis (e.g., alcohol oxidation), thereby enabling mediator recovery by dialysis or membrane filtration without the need for chromatographic separation (Figure 5a).^59–61^ Although the catalytic current densities decreased markedly with increasing *M*_n_, the Faradaic efficiency remained consistently high. Overall, the researchers identified an optimal *M*_n_ window (approximately a few kDa) that balances fast electrocatalysis with practical recyclability, providing design guidance for sustainable redox mediators.

**Figure 5:**
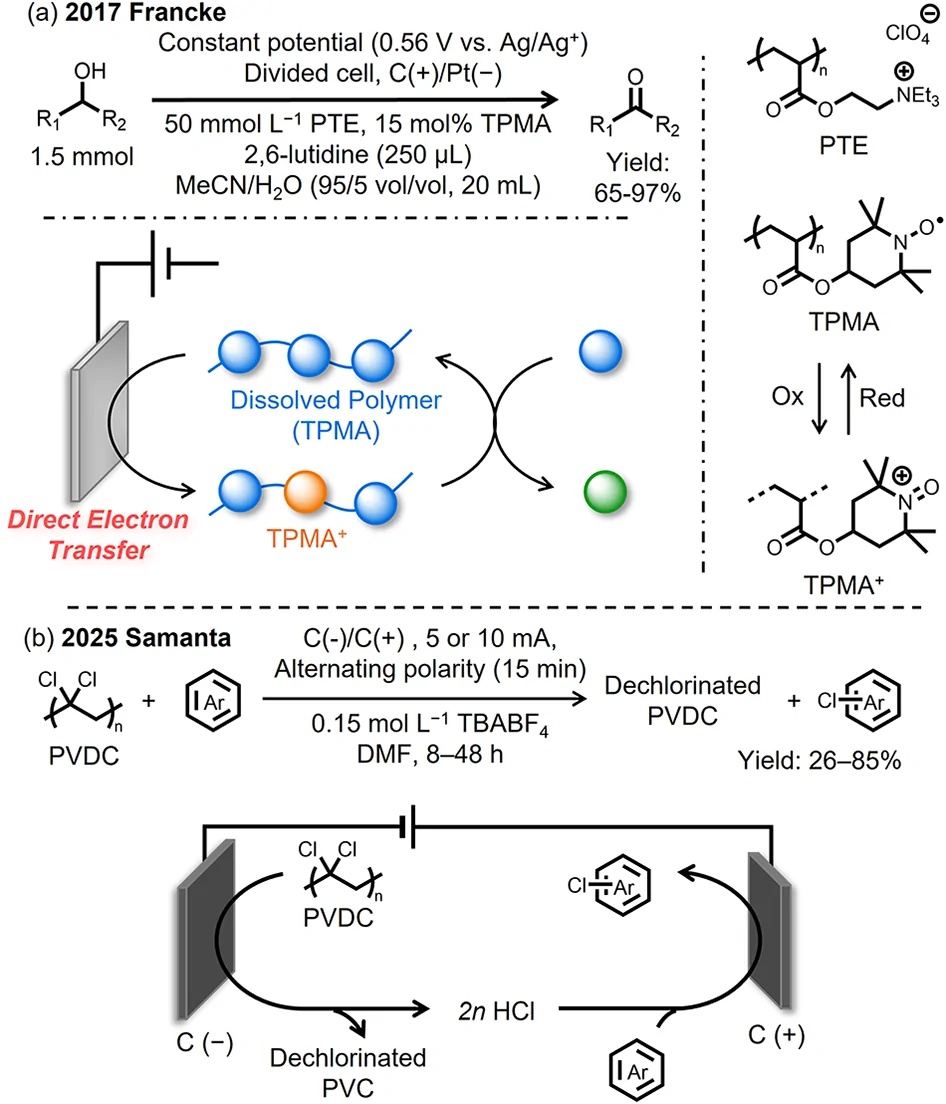
(a) Schematic representation of TPMA-mediated anodic oxidation of alcohols and chemical structures of poly­(trialkylammonium ethyl methacrylate) and TPMA. Adapted with permission from ref 60. Copyright 2018 Wiley-VCH. (b) Electrochemical reductive dechlorination of PVDC in undivided cell and use of released chloride ions for anodic chlorination of arenes. Adapted with permission from ref 63. Copyright 2025 Wiley-VCH.

Fuchigami and co-workers reported polystyrene-supported iodobenzene (PSIB) as a recyclable heterogeneous solid-phase mediator for indirect anodic fluorination of organosulfur compounds.^62^ In contrast to TPMA, which is directly oxidized at the electrode, PSIB is activated indirectly via electrogenerated Cl^+^. In this double mediatory system, anodic oxidation of Cl^–^ generates Cl^+^, which reacts with the iodobenzene moiety of PSIB to form a hypervalent [(chloro)­(fluoro)­iodo]­benzene species that subsequently fluorinates the substrate. Because PSIB is insoluble in the ionic liquid electrolyte, the reactive hypervalent iodine species generated is not quenched at the cathode, enabling the use of a simple undivided cell. Furthermore, PSIB could be readily recovered by filtration and reused for at least ten consecutive runs without significant loss of mediatory activity.

Samanta et al. reported the direct reductive dechlorination of polyvinylidene chloride (PVDC) using an undivided cell (Figure 5b).^63^ The cathodic reduction of PVDC released chloride ions, and repeated electrolytic cycles removed up to 98% of the chlorine from the PVDC backbone. Remarkably, this approach proved highly robust against commercial additives, enabling the direct dechlorination of real plastic waste, such as pharmaceutical blister films and multilayer food packaging. Notably, the released chloride ions can be further used at the anode for the oxidative chlorination of arene substrates, demonstrating the potential of paired electrolysis for valorizing waste-derived chlorine.

## 4 *e*PF of Polymers via Indirect Activation Using Redox Mediators

In this mode, polymers are indirectly activated via redox mediators^64^ that are oxidized or reduced at the electrode and subsequently transfer electrons to the polymer substrate. This approach is particularly effective when direct electron transfer between the electrode and the polymer is inefficient, such as in the case of high molecular weight or poorly conducting polymers. MacNeil et al. compared direct and indirect electrolysis of PVC and reported that the indirect approach is more effective for high-molecular-weight samples (Figure 6a).^65^ In their system, di­(2-ethylhexyl)­phthalate serves as a redox mediator for the reductive dechlorination of PVC, enabling the efficient release of chloride. Subsequently, the released chloride is anodically oxidized to reactive chlorine species that chlorinate arene substrates, demonstrating the repurposing of PVC waste as a chlorine source and highlighting a potentially more sustainable reaction platform. Similarly, MacNeil and colleagues demonstrated that PVC electrolysis yields high Faradaic efficiencies for both chlorine evolution (>80% yield) and base generation (>90% yield), providing a strong foundation for future reaction engineering.^66^

**Figure 6:**
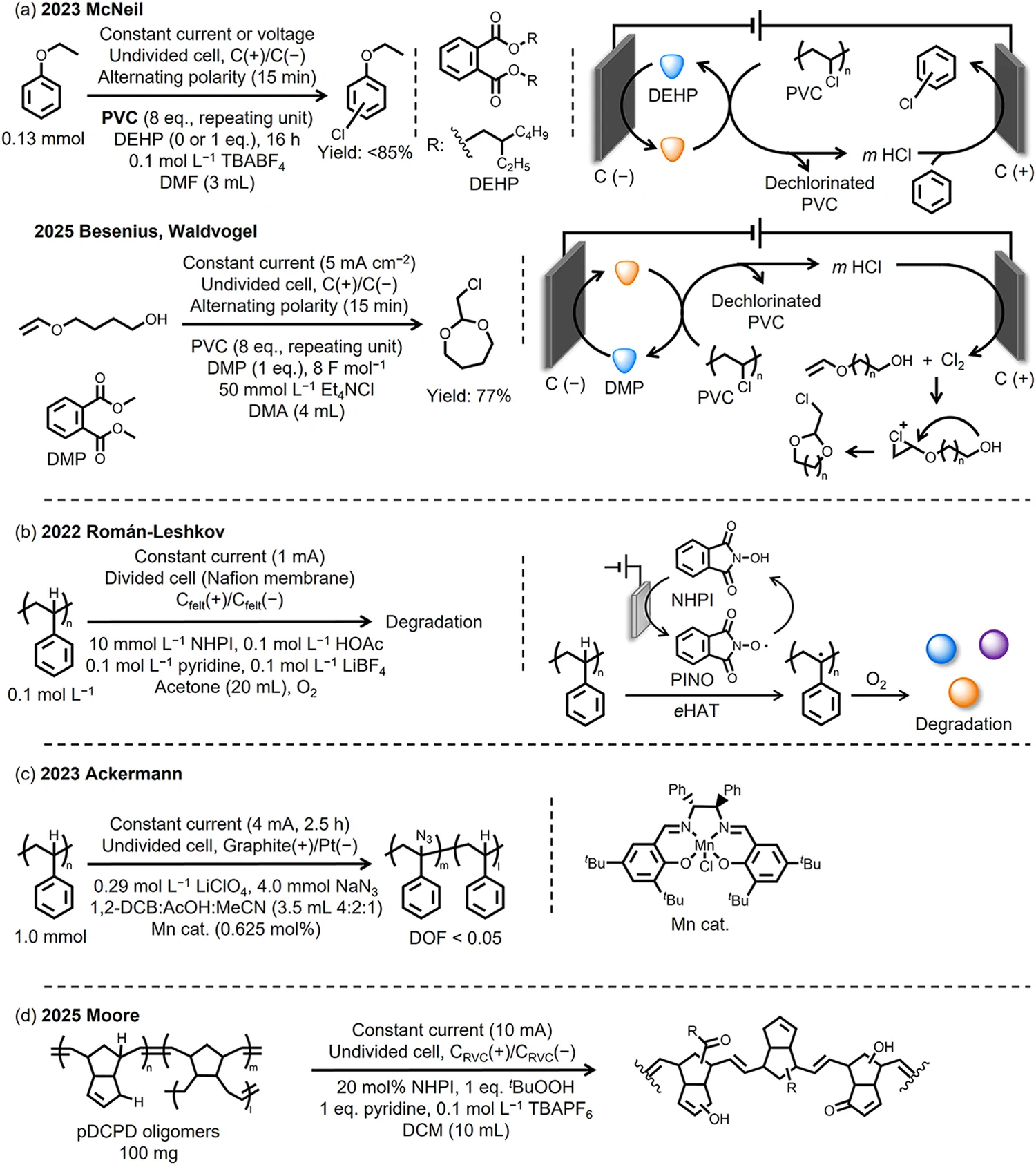
(a) Reaction scheme of cathodic dechlorination of PVC and anodic chlorination of arenes and vinyl ethers. Adapted with permission from ref 67. Copyright 2026 Wiley-VCH. (b) Anodic degradation of polystyrene mediated by the NHPI/PINO system and its proposed *e*HAT mechanism. Adapted with permission from ref 69. Copyright 2022 Wiley-VCH. (c) Anodic azidation of polystyrene using Mn electrocatalyst. (d) Dual C–H functionalization of DCPD oligomers via NHPI/PINO-mediated *e*HAT.

Furthermore, Besenius, Waldvogel, and colleagues demonstrated the electrochemical chlorination of vinyl ethers through a process involving the dimethyl phthalate-mediated cathodic dechlorination of PVC (Figure 6a).^67^ This method achieved up to 94% dechlorination for PVC and a 77% GC yield for chlorinated vinyl ether. Notably, this paired electrolysis strategy exhibited high tolerance to commercial plastic additives, enabling the direct upcycling of PVC waste streams into valuable monomer precursors.

*N*-Hydroxyphthalimide (NHPI) can also serve as a redox mediator that undergoes anodic oxidation to generate a phthalimide-*N*-oxyl (PINO) radical (Figure 6b). PINO mediates hydrogen-atom transfer (HAT) from organic substrates, forming a carbon-centered radical and regenerating NHPI in the catalytic cycle. The NHPI/PINO redox couple is typically tuned by adding a base, which shifts the oxidation potential to less positive values; thus, bases are commonly included in the electrolyte.^68^ Román–Leshkov and colleagues reported the electrochemical degradation of polystyrene induced by electrochemical HAT (*e*HAT) (Figure 6b).^69^ The electrogenerated PINO radical facilitated benzylic HAT to generate a carbon radical that was trapped by O_2_ to form a peroxide and then fragmented into oxygenated C–C scission products. Because polymer oxidation occurs through the mediator, the required anodic potential is substantially lowered (∼1.2 V) compared with direct polymer oxidation, thereby enabling milder and more selective bond activation.

Ackermann et al. demonstrated regioselective C­(sp^3^)–H azidation utilizing a manganese electrocatalyst.^70^ By performing constant-current electrolysis on dissolved polystyrene in the presence of a sterically bulky salen-type manganese complex, NaN_3_ as the azide source, and lithium perchlorate as the supporting electrolyte, the researchers successfully obtained azidated polystyrene (Figure 6c). Mechanistically, the reaction proceeded via the anodic oxidation of a Mn­(III) complex to a reactive Mn­(IV) diazide species, which then activated the benzylic C–H bond via *e*HAT for subsequent azide introduction. Although the DOF was relatively low at a few percent, this protocol was also applicable to inert polyolefins, achieving slight but distinct azidation even on polyethylene and polypropylene. The incorporated azide groups served as versatile synthetic handles for further postmodification via click chemistry, enabling further diversification of polymer functionality.

Moore and colleagues reported a paired electrolysis strategy that enables one-step dual C–H functionalization of deconstructed oligo-dicyclopentadiene waste generated during carbon–fiber composite recycling (Figure 6d).^71^ Through NHPI/PINO-mediated HAT under mild electrolysis conditions, two key backbone functionalizations, namely, α,β-unsaturated ketones (C–O formation) and phthalimides (C–N bond formation), were achieved (Figure 6d). Competition studies demonstrated predominant tertiary allylic C–H activation along the branched backbone. The resulting difunctionalized oligomers were converted into covalently adaptable networks with dynamic imine/oxime linkages, enabling reprocessing/self-healing. Furthermore, amine-triggered programmed deconstruction was demonstrated, yielding highly recyclable materials directly from waste oligomers.

## 5 *e*PF of Polymers via Electrochemically Generated Reactive Species

In this mode, electrochemically generated reactive species subsequently react with dissolved polymers, providing *e*PF pathways without direct electron transfer between the electrode and the polymer. This approach is broadly applicable to nonconjugated polymers and enables diverse chemical transformations through the choice of electrolyte composition. Yoshida, Nokami, and colleagues demonstrated the anodic electrophilic aromatic substitution of polystyrene with diarylcarbenium ions (DOF: ∼ 0.80, Figure 7b).^72,73^ Diarylcarbenium ions were generated and accumulated at – 78 °C via the oxidation of trimethylsilyl–diarylmethane using the cation pool method. Subsequently, polystyrene was added to the solution, and the mixture was stirred at 0 °C for 1.5 h. Functionalization selectively proceeded at the 4-position of polystyrene due to the steric hindrance of the diarylcarbenium intermediate.

**Figure 7:**
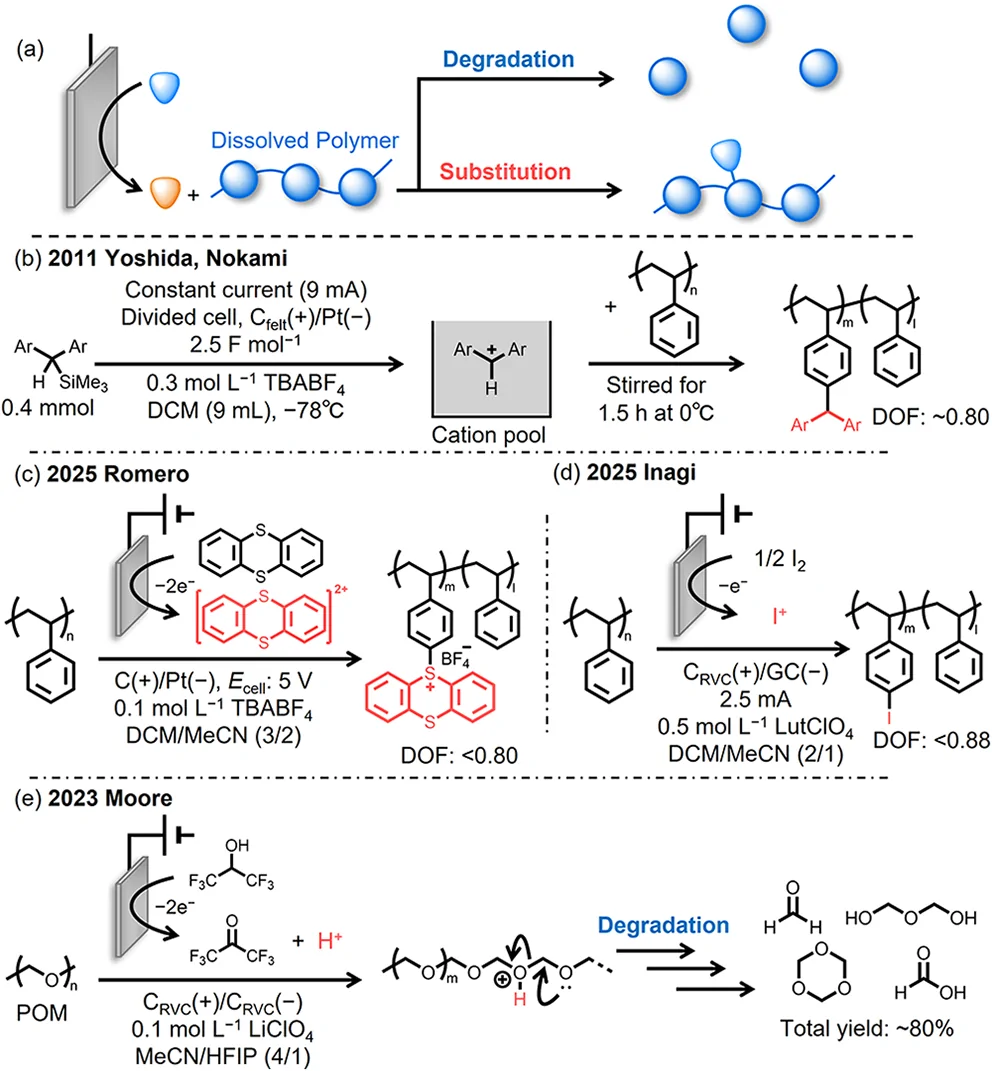
(a) Schematic representation of *e*PF via electrochemically generated reactive species. (b) Electrophilic aromatic substitution of polystyrene with diarylcarbenium ions using the cation pool method. (c) Electrochemical thianthrenation of polystyrene. (d) Anodic iodination of polystyrene. Adapted with permission from ref 4. Copyright 2025 American Chemical Society. (e) Depolymerization of POM via electrochemically generated acid through anodic oxidation of HFIP. Adapted with permission from ref 75. Copyright 2023 Springer Nature.

Naumann and colleagues demonstrated the electrochemical C–H thianthrenation of commodity polystyrene using simple thianthrenes to access thianthrenium (sulfonium) polyelectrolytes with a controllable DOF (Figure 7c).^74^ The electrochemically generated thianthrene radical cation promoted C–H thianthrenation on the aryl groups of polystyrene via electrophilic aromatic substitution. Notably, the resulting arylthianthrenium unit enabled selective reductive photocleavage, allowing the defunctionalization and recovery of polystyrene, demonstrating the potential for circular polymer modification.

Inagi, Wang, and colleagues reported the mild electrochemical postmodification of polystyrene via aromatic C–H iodination, where the anodically generated cationic iodine species (I^+^) served as a clean electrophile for electrophilic aromatic substitution (Figure 7d).^4^ Cyclic voltammetry (CV) analysis supported a two-step pathway: first, iodine was oxidized at the anode to produce iodine cations; then, these cations iodinated polystyrene via electrophilic aromatic substitution. Their method was compatible with polystyrene over a wide molecular weight range and could be applied directly to real polystyrene waste, including expandable polystyrene and plastic spoons.

Zhou, Rodríguez-López, and Moore reported the first electrochemical depolymerization of highly crystalline polyoxymethylene (POM, Delrin) to repolymerizable monomeric products (formaldehyde/1,3,5-trioxane) under ambient conditions (Figure 7e).^75^ Solvent screening identified a MeCN/HFIP system as uniquely effective, where HFIP solvates and disrupts the crystalline acetal chain packing, enabling rapid deconstruction. Mechanistic experiments, including CV, divided-cell studies, model compound evaluations, and control reactions, support an electrochemically generated acid-mediated depolymerization mechanism in which anodic oxidation of HFIP generates protons that trigger chain degradation. The approach was demonstrated on postconsumer Delrin waste at the gram scale, highlighting an electricity-driven route toward circular recycling of an otherwise difficult-to-recycle engineering thermoplastic.

## 6 Conclusions and Outlook

*e*PF has emerged as a powerful and sustainable platform for polymer modification, offering precise control over the DOF through simple tuning of electrochemical parameters. The mechanistic modes presented in this review, namely, direct activation via solid-to-solid and solid-to-solution electron transfer and indirect activation via redox mediators or electrochemically generated reactive species, collectively demonstrate the versatility and broad applicability of *e*PF across diverse polymer systems.

Despite these advances, several challenges remain. Overoxidation and undesired chain degradation can compromise the structural integrity of the polymer backbone, while competing electrode processes such as solvent or electrolyte oxidation reduce current efficiency. Mass transport limitations arising from the low diffusivity of high molecular weight polymers represent another key challenge, which can be mitigated by stirring-induced convection or flow electrochemical systems. Addressing these limitations through careful optimization of electrochemical conditions and reactor design will be essential for broadening the scope of *e*PF to new polymer systems.

For CPs, the choice between solid-state and solution-phase reaction environments is dictated by the solubility of the oxidized polymer species; judicious selection of appropriate reaction conditions enables precise tuning of optical, structural, and electronic properties. These advances are expected to accelerate the development of high-performance materials for organic electrochemical transistors, bioelectronic sensors, and related devices.

Beyond functional materials, *e*PF provides promising pathways for the chemical valorization of commodity plastics, such as polystyrene, PVC, and PVDC, contributing to the realization of a circular plastics economy. Future efforts should focus on transitioning these methodologies from well-defined polymer samples to real-world, multicomponent plastic waste streams, which will require careful attention to electrode fouling, macromolecular mass transport, and reactor design. The integration of continuous electroflow systems and paired electrolysis strategies will be essential for scalable implementation. As renewable energy sources increasingly power these electrochemical processes, the sustainability profile of *e*PF will be enhanced, positioning it as a cornerstone methodology for next-generation polymer chemistry.
